# Mentoring in research: development of competencies for health professionals

**DOI:** 10.1186/s12912-023-01411-9

**Published:** 2023-07-26

**Authors:** Regina Claudia da Silva Souza, Mariana Davies Ribeiro Bersaneti, Wellington Pereira dos Santos Yamaguti, Wania Regina Mollo Baia

**Affiliations:** Hospital Sírio Libanês, São Paulo, Brazil

**Keywords:** Mentors, Research, Health personnel

## Abstract

**Background:**

Mentoring programmes in health research are beneficial for both mentors and mentees and are essential for the development of the next generation of research leaders. This study describes the self-assessment of research skills in health professionals participating in a research mentoring programme and determines the correlation between the participants’ self-assessment of research skills and professional characteristics.

**Method:**

This was a quasi-experimental, time-series study conducted in a Brazilian tertiary hospital. Thirty-five health professionals holding a master’s or PhD degree were included. The participants answered a survey in which they self-assessed their research skills distributed into eight domains, with one group responding before training and another group responding after training. The level of significance was set at 5% (*p* < 0.05).

**Results:**

Those who received training scored better in research skills related to two domains: critical analysis of the literature and identification of appropriate research methods (*p* = 0.0245).

**Conclusion:**

Trained professionals performed better in the domains of critical thinking and knowledge and management of steps in the research process.

## Background

The literature highlights the need to promote a culture of research support and encouragement in health organisations through the engagement of health professionals in this process. Institutional research culture is based on the reiterated investigative behaviour of research professionals, organisational capacity to produce research, and an infrastructure that facilitates the development of high-quality studies [1].

Those responsible for policy planning and strategy implementation must identify resources to promote research among healthcare professionals and the development of a research culture. These aspects are fundamental to improving evidence-based practice (EBP) and research competencies among professionals [2]. Some principles are essential in the development of an institutional research culture, such as improving skills and trust, establishing bonds and partnerships, training researchers who are close to the practice, improving appropriate dissemination, investing in infrastructure and building elements of sustainability and continuity. However, an institutional research culture is affected by individual and organisational factors, among which are multidisciplinary research groups [3].

Plans to develop health professionals’ research skills are relevant because they affect a strong institutional culture that promotes an environment in the organisation to produce knowledge and interaction and collaboration with other organisations [4]. Therefore, knowing and evaluating the development of research skills in health professionals and of successful strategies to achieve high performance in research are important institutional planning aspects.

The mentoring programme in research may be a successful strategy in hospitals to improve the use of research in clinical practice. Mentoring can be defined as a relationship between two people, in which the mentor is an individual with experience in developing the skills of the other. Mentoring relationship includes professional development and emotional support for developing skills and self-confidence [5].

In this context, mentors are professionals with experience in conducting and teaching research and function as advisers for health professionals involved in assisting the development of research [6, 7]. Their role is to guide and supervise healthcare professionals in research projects. Further, given their experience, mentors can also contribute to the training of other professionals while helping them with the various problems and choices presented.

A study conducted in Magnet® hospitals indicated that 96% of hospitals that participated in the survey reported there were research mentors available to supervise nursing research [8]. In a Magnet Journey hospital in Brazil, clinical nurses reported having positive beliefs about the benefits of nurse-led research, especially regarding the impact of research on the image of the institution, development of teamwork, and patient care [9].

Mentoring programmes in healthcare are beneficial for mentors, mentees, and the involved institutions, and are essential for the development of the next generation of research leaders. These programmes are recognised in the USA and other countries as an important academic strategy to support the new generation of research nurses [5]. They have recently been implemented in the in American hospitals that are on the Magnet Journey to help professionals develop different skills. Thus, this training model was chosen to prepare young researchers to help the development of other professionals, increase knowledge production and transfer, and engage mentors in advancing their careers as researchers.

However, studies are necessary to demonstrate the benefits of these programmes and their return on investment [10] through the examination of not only the validity of the assessment measures but also the wide range of research skills identified for summative and formative assessment. These findings have important implications for the education of healthcare professionals and contribute to the development of the next generation of clinical and translational researchers [11].

Mentoring programmes in health research can be improved by using key competencies to define the set of skills required for effective mentoring, determine the research mentors’ training needs, and facilitate the building of the institutional capacity to support them, which includes resources, institutional guidelines, and financial and administrative support for mentorship [10]. Therefore, a competency in research profile developed by Wester et al. [12] was selected to assess a structured programme of research mentorship to train health professional researchers in research skills.

The objectives were to describe the self-assessment of research skills in health professionals participating in a research mentoring programme and determine the correlation between the participants’ self-assessment of research skills and sociodemographic characteristics.

## Method

### Study design

This was a quasi-experimental, time-series study conducted between September 2020 and June 2021 in a major tertiary hospital in the municipality of São Paulo, Brazil. The hospital has 523 beds and has been accredited and recognised by major certifying entities in Brazil. The convenience sample included 35 healthcare professionals with a master’s or PhD degree.

### Study participants

The inclusion criteria were healthcare professionals with a master’s or PhD degree who agreed to participate in a programme of research mentorship. The convenience sample was 35 professionals and included nurses, nutritionists, pharmacists, physical therapists, and psychologists. The professionals who did not participate in 100% of the training activities were excluded.

### Legal ethical aspect

The study was conducted in accordance with human research guidelines and regulations. All participants in this study signed an informed consent form (ICF) and were instructed on the main aspects of the study and their right to refuse to participate or withdraw from the study at any time. The study was approved by the Research Committee of the institution where the study was conducted, under no. 4.205.400.

### Data collection

The training was developed over five virtual meetings, with a total duration of 20 h, and was conducted by professionals with experience in research. Each meeting lasted four hours and the content included the development of a research question, types of study design, basic statistics (variables and main tests), reading a scientific paper, creating a database, and procedures for submitting research projects. The meetings were held via a remote online platform using team-based learning (TBL), group discussions, and lectures. In addition, a platform (Canvas) was used for the repository of support material (articles and content), with access granted to training participants. Meanwhile, the topics addressed were related to a profile of competencies in research [12]. Finally, activities focusing on basic research skills were performed, and, foreseeing the prolongation of the program, continuing education through bimonthly workshops for the development of advanced competencies was planned.

Data were collected through a survey, which recorded professional and sociodemographic information and self-assessment of research skills. The Research skills self-assessment questionnaire prepared by the researchers included 149 items organised into eight domains, and the Likert scale was used for responses, with scores from 1 to 5 corresponding to strong disagreement and strong agreement, respectively. The domains of the competencies were organized into critical thinking and knowledge, management of the steps in the research process, data collection and analysis, communication of research findings, ethical and professional competence, research assessment, relational aspects, and continuing education, based on the reference by Wester et al. in a study conducted on the topic that validated the profile of research competencies using the Delphi method with 10 panellists [12]. The questionnaire had a Cronbach’s alpha of 0.99. The participants’ answers were analyzed according to the self-assessed proficiency level for the competence profile of a researcher.

The electronic survey containing the data collection instrument was sent via e-mail to the participants before and three months after the training, which was open for answers only after the participants had signed the informed consent document. This questionnaire was chosen due to the non-availability of a validated instrument in Brazil to assess research skills in health professionals. The professionals who answered the questionnaire were divided into two independent groups: the untrained group and the trained group. The design of this time-series study considered the untrained group as the control; there was no randomization between the groups.

The outcome variable was the self-assessment of the profile of research competencies. The sociodemographic and professional variables included age, duration of professional experience, duration since the conclusion of master’s or PhD degree, professional category, title, publication experience, publication of a thesis, experience in supervising scientific projects, experience with funding agencies, other institutional research affiliations, and number of publications after the conclusion of the master’s or PhD program.

### Statistical analysis

The data were analysed using the Statistical Package for the Social Sciences® (SPSS) 23.0. The qualitative variables were expressed as relative (%) and absolute (n) frequencies and were compared between the groups with and without training using the chi-square test. Fisher’s exact and likelihood ratio tests were used when necessary.

The normality of the quantitative variables was evaluated by means of the Kolmogorov–Smirnov test. The quantitative variables were expressed as median and interquartile range (IQR) and analysed according to training using Student’s t-test or the Mann–Whitney test. Spearman’s rank coefficient of correlation was used to correlate the sociodemographic variables with the domains of the profile of competence in research. The level of significance was set at 5% (*p* < 0.05) in all statistical tests.

## Results

The study comprised a total sample of 35 healthcare professionals. Of these, 18 and 17 professionals conducted the self-assessment of research competencies before and three months after the training of the mentoring program, respectively. The data in Table 1 show that the groups had similar sociodemographic characteristics and that training was the main difference between them. In the sample, 45.7% (16) of the individuals were nurses, 25.7% (9) were nutritionists, 22.9% (8) were physical therapists, and 5.7% (2) were pharmacists. The median age was 37 years, 88.6% (31) had a master’s degree, 11.4% (4) had a PhD, and 91.4% of the participants had experience with scientific publication. The mean length of professional experience was 13.57 ± 4.6 years. Regarding research guidance, 60% (21) claimed to have this experience, 65.7% (23) of professionals published between 1 and 3 articles after the graduation, 17% (6) published between four and six articles and 17.1% (6) published none.

**Table 1:** Sociodemographic and professional characteristics of the healthcare team

	**Groups**		**Total**	***p*-value**
	**Without training** **(*n* = 18)**	**With training** **(*n* = 17)**		
**Age, median (IQR)**	36.5 (32–40)	37 (33–39)	37 (32–40)	0.929^a^
**Duration of professional experience, median (IQR)**	13 (10–18)	12 (10–16)	13 (10–18)	0.846^a^
**Time since master’s or PhD, median (IQR)**	4 (2–5)	4 (2–6)	4 (2–6)	0.751^b^
**Profession, n(%)**				
Nurse	8 (44.4)	8 (47.1)	16 (45.7)	1.000^c^
Other	10 (55.6)	9 (52.9)	19 (54.3)	
**Title, n(%)**				
Master’s	16 (88.9)	15 (88.2)	31 (88.6)	1.000^d^
PhD	2 (11.1)	2 (11.8)	4 (11.4)	
**Publication experience, n(%)**				
Yes	15 (83.3)	17 (100.0)	32 (91.4)	0.228^d^
No	3 (16.7)	0 (0.0)	3 (8.6)	
**Publication of thesis, n(%)**				
Yes	11 (61.1)	12 (70.6)	23 (65.7)	0.554^c^
No	7 (38.9)	5 (29.4)	12 (34.3)	
**Experience supervising scientific projects, n(%)**				
Yes	11 (61.1)	10 (58.8)	21 (60.0)	0.890^c^
No	7 (38.9)	7 (41.2)	14 (40.0)	
**Experience with funding agencies, n(%)**				
Yes	9 (50.0)	8 (47.1)	17 (48.6)	0.861^c^
No	9 (50%)	9 (52.9%)	18 (51.4%)	
**Other institutional research affiliation, n(%)**				
Yes	2 (11.1%)	4 (23.5%)	6 (17.1%)	0.401^d^
No	16 (88.9%)	13 (76.5%)	29 (82.9%)	
**Number of publications after the conclusion of the master’s or PhD program, n(%)**				
0	4 (22.2%)	2 (11.8%)	6 (17.1%)	0.706^e^
Between 1 and 3	11 (61.1%)	12 (70.6%)	23 (65.7%)	
Between 4 and 6	3 (16.7%)	3 (17.6%)	6 (17,1%)	

*IQR* Interquartile range

^a^t-test

^b^Mann-Whitney test

^c^Chi-square test

^d^Fisher’s exact test

^e^Likelihood ratio test

There were differences between the groups in the “critical thinking and knowledge” and “management of the steps in the research process” competencies. This indicated that the group that received training related to critical analysis of the literature and identification of appropriate research methods had higher scores than the group that did not. The remaining domains did not differ between the groups, as shown in Table 2.

**Table 2:** Self-assessment of research competencies by healthcare professionals

Domain (median, IQR)	Group		Total	*p*-value
	**Without training** **(*n* = 18)**	**With training** **(*n* = 17)**		
**Critical thinking and knowledge**	21.0 (15–37)	32.0 (29–46)	31.0 (18–42)	**0.045**^**a**^
**Management of steps in the research process**	4.5 (1–14)	20.0 (7–23)	9.0 (2–21)	**0.024**^**b**^
**Data collection and analysis**	7.5 (5–13)	15.0 (11–21)	12.0 (6–18)	0.111^a^
**Communication of research findings**	11.5 (3–16)	15.0 (14–19)	15.0 (4–17)	0.097^b^
**Ethical and professional competence**	7.5 (6–8)	7.0 (5–8)	7.0 (6–8)	0.526^b^
**Research assessment**	5.0 (2–6)	6.0 (5–6)	5.0 (4–6)	0.147^b^
**Relational aspects**	5.0 (3–5)	5.0 (4–5)	5.0 (4–5)	0.301^b^
**Continuing education**	4.0 (4–4)	4.0 (4–4)	4.0 (4–4)	0.481^b^

*IQR* Interquartile range

*p*-values in bold are statistically significant

^a^t-test

^b^Mann-Whitney test

The sociodemographic and professional characteristics of the mentors were correlated with the domains of competencies, and a significant correlation was observed between duration of professional experience and “communication of research findings”. The longer the professional experience, the better the self-evaluation related to knowledge of scientific writing. Duration of professional experience was also significantly correlated with the domains “research assessment” and “relational aspects” (Table 3).

**Table 3:** Correlation between the domains of research competencies and duration of professional experience

**Domains of competencies**	**Duration of professional experience**
**Critical thinking and knowledge**	
R^a^	0.320
*p*-value	0.056
**Management of steps in the research process**	
R^a^	0.310
*p*-value	0.067
**Data collection and analysis**	
R^a^	0.310
*p*-value	0.073
**Communication of research findings**	
R^a^	0.340
*p*-value	**0.043**
**Ethical and professional competence**	
R^a^	0.260
*p*-value	0.134
**Research assessment**	
R^a^	0.410
*p*-value	**0.013**
**Relational aspects**	
R^a^	0.350
*p*-value	**0.037**
**Continuing Education**	
R^a^	0.160
*p*-value	0.373

*p*-values in bold are statistically significant

^a^Spearman’s Coefficient of Correlation

## Discussion

The development of a culture of research and the engagement of health professionals in research is a great challenge for institutions. The research culture improves the outcomes of care because it results in clinical decisions based on knowledge. The findings of the present study show that self-assessment of trained professionals regarding research competencies was better in some domains that who did not do training. These findings confirm the benefits of the programme and its importance for the development of future researchers. Nursing scientist development programs, which can be applied to other professionals, are essential to improve and acquire new research skills; thereby addressing the shortage of scientists in nursing, as well as in other health areas [13]. In a similar project conducted by the Memorial Sloan Kettering Cancer Center, scientific production was found to be significant among nurses who underwent training, namely through participation in meetings and publication in journals [1].

The participants in the present study were young and recently trained as researchers. Most had yet to attain a doctoral degree. Considering that the development of a researcher’s competency profile in healthcare is a continuous and long-life process that requires dedication and academic involvement with peers and care teams [14], the better performance of the trained group in two domains of competencies in research may be considered a positive result, even if it cannot be exclusively attributed to the training activity.

Notably, core competencies in research are necessary to structure training programmes for the development of research skills, and the recommended strategies include mentoring programmes [14]. The group that received training performed better in the domains “critical thinking and knowledge” and “management of steps in the research process,” which was expected because the topics addressed during the training were related. The level of proficiency in the other domains showed the group’s need to develop certain competencies over time. As such, the mentoring programme involved in this study organizes regular meetings on different topics according to the mapped competencies. According to core competencies in research, these domains are fundamental for the professional development of a researcher [14]

Research competencies for healthcare professionals are constantly evolving because new research questions emerge; further, new methods are developed, and the transdisciplinary nature of the field creates new needs. Therefore, competencies are proposed as suggestions to training programmes that can innovate on preparing future researchers in healthcare services [14]. In this study, the mentoring programme involved health professionals because transdisciplinarity is an essential element in the development of researchers and promotes the production of knowledge.

Among professional characteristics, duration of professional experience was correlated with the domains “communication of research findings”, “research assessment”, and “relational aspects”. Research communication encompasses the ability to effectively communicate the process, findings, and implications of the research, and is closely related to the relational aspects because both involve the ability to work in collaboration with teams within the same field and from different fields, and/or with stakeholders. These domains are extremely relevant for the development of researchers. More experienced professionals can be inferred to be more dedicated to the development of these skills because they understand that these aspects are relevant for their careers [14]. These results contradict the assessment of self-efficacy in research, wherein nurses with less ability to draft papers were those with longer experience since graduation [9]; however, teaching favored overall research competence, including the ability to search for information and use evidence-based practices and theoretical knowledge [9].

In a systematic review on the topic, training was consistently associated with self-assessed competency but had a small relationship with objective competency measurements [11]. The studies included in this systematic review addressed self-assessment and objective assessment and revealed statistically significant results regarding the self-assessed competencies and most of the abilities or domains after the research training programmes [11]. The review authors recommend assessing research competencies using objective and subjective measurements and pre- and post-training testing to analyse the effects of training programmes [11].

The limitation of this study was that the two groups of assessed mentors were independent and not paired. It was thus not possible to establish a causal relationship between training and the development of competencies, despite the participants’ sociodemographic and professional characteristics being similar.

## Conclusion

The healthcare professionals who received training performed better in the “critical thinking and knowledge” and “management of steps in the research process” domains. The period of professional experience was correlated with communication of research findings, research assessment, and relational aspects. Further studies are necessary to demonstrate the strength of the intervention in the development of future researchers, who are essential for the sustainability of healthcare services and for the development of instruments to assess the competency profile of a researcher.

## Data Availability

The datasets used and/or analysed during the current study are available from the corresponding author on reasonable request.

## Abbreviations

EBP: Evidence-based practice
ICF: Informed consent form
TBL: Team-based learning
